# A Retrospective Exploration of Targeted Maintenance Therapy in Advanced Colorectal Cancer: Based on the Background of Chinese Patient Assistance Program

**DOI:** 10.3389/fonc.2020.00522

**Published:** 2020-04-23

**Authors:** Hanguang Hu, Xue Liu, Wen Cai, Dehao Wu, Junxi Xu, Ying Yuan

**Affiliations:** ^1^Departments of Medical Oncology, The Second Affiliated Hospital, Zhejiang University School of Medicine, Hangzhou, China; ^2^Cancer Institute (Key Laboratory of Cancer Prevention and Intervention, China National Ministry of Education), The Second Affiliated Hospital, Zhejiang University School of Medicine, Hangzhou, China

**Keywords:** colorectal cancer, cetuximab, bevacizumab, maintenance therapy, KRAS wild-type

## Abstract

**Background:** Maintenance therapy with bevacizumab (Bev) in patients with colorectal cancer (CRC) provides progression-free survival (PFS) benefits. However, the role of maintenance therapy with an anti-EGFR monoclonal antibody has not been established.

**Methods:** Eligible CRC patients were assigned to maintenance therapy with cetuximab (Cet; Cet group) or Bev (Bev group). PFS, the duration of maintenance therapy, and safety were analyzed. Cox multivariate regression analyses were performed to determine independent prognostic factors.

**Results:** A total of 143 eligible patients were assigned to the Cet (*n* = 79) or Bev (*n* = 64) groups. In the Cet group, all patients had KRAS wild-type. The baseline characteristics were well-balanced between the two groups, except for a higher percentage of patients with a left-sided primary tumor in the Cet group than in the Bev group (86.1 vs. 62.5%, *P* < 0.0001). The median PFS was not significantly different between the Cet group and the Bev group: 5.9 months (95% CI 2.30–9.50) vs. 7.0 months (95% CI 3.69–10.31) (HR 1.17, 95% CI 0.77–1.79, *P* = 0.45). The median duration of maintenance therapy in the Cet group was shorter than that in the Bev group: 4.0 months (95% CI 1.94–5.99) vs. 4.8 months (95% CI 2.68–6.98) (HR 0.90, 95% CI 0.61–1.33; *P* = 0.59). The subgroup analyses showed that the median PFS for the first maintenance therapy and the second maintenance therapy were 3.2 months (95% CI 1.69–4.78) and 5.2 months (95% CI 1.58–8.83), respectively (HR 0.89, 95% CI 0.44–1.81; *P* = 0.75).

**Conclusions:** This study suggests that maintenance therapy with Cet or Bev can be considered an appropriate option following induction chemotherapy for selected patients with advanced CRC. Multiple maintenance therapy seems to confer survival benefits in advanced CRC. Maintenance therapy with Cet after first-line induction chemotherapy seems to be associated with greater survival benefits.

## Introduction

Colorectal cancer (CRC) is the third most frequent cancer and the second leading cause of death from cancer worldwide (1). The prognosis of patients with CRC is poor (2, 3); that is, more than half of patients will ultimately develop metastases (4–6). The median overall survival (OS) in patients with mCRC is ~30 months (7) due to the availability of several chemotherapy drugs and targeted drugs as well as the development and popularization of multidisciplinary treatment models (8–10).

At present, targeted drugs for mCRC consist of anti-epidermal growth factor receptor (anti-EGFR) monoclonal antibody and anti-vascular endothelial growth factor (anti-VEGF) monoclonal antibody. The former consists of cetuximab (Cet) or panitumumab, while the latter is bevacizumab (Bev). The addition of Cet or Bev has led to significant benefits in terms of OS or progression-free survival (PFS) for selected patients with CRC (11).

Improving the OS of patients with CRC also extends their time on treatment and the associated side effects (12). Therefore, several studies have been conducted to evaluate new strategies for the release of such cases without compromising survival (13, 14). Randomized trials have shown that maintenance therapy with Bev is superior to intermittent treatment or continuous chemotherapy in patients with mCRC (9, 15–17). The maintenance regimens currently used are fluoropyrimidines alone, Bev alone, or fluoropyrimidines with Bev (18). Clinical trials have shown that maintenance therapy with Cet can be safely added to intermittent chemotherapy and that is confers similar survival benefits as continuous chemotherapy but is less toxic. However, this treatment strategy is still controversial (18).

Moreover, some patients have received maintenance therapy with Cet in clinical practice because of the medical insurance in Zhejiang Province and the Chinese Patient Assistance Program. Due to the high cost of targeted therapeutic drugs, after patients buy a certain amount of drugs, they are provided with free drugs that are used until the disease progresses. Therefore, we conducted this retrospective study to investigate the efficacy of maintenance therapy with Cet or Bev.

## Patients and Methods

### Data Source and Preliminary Analysis

**Figure 1:**
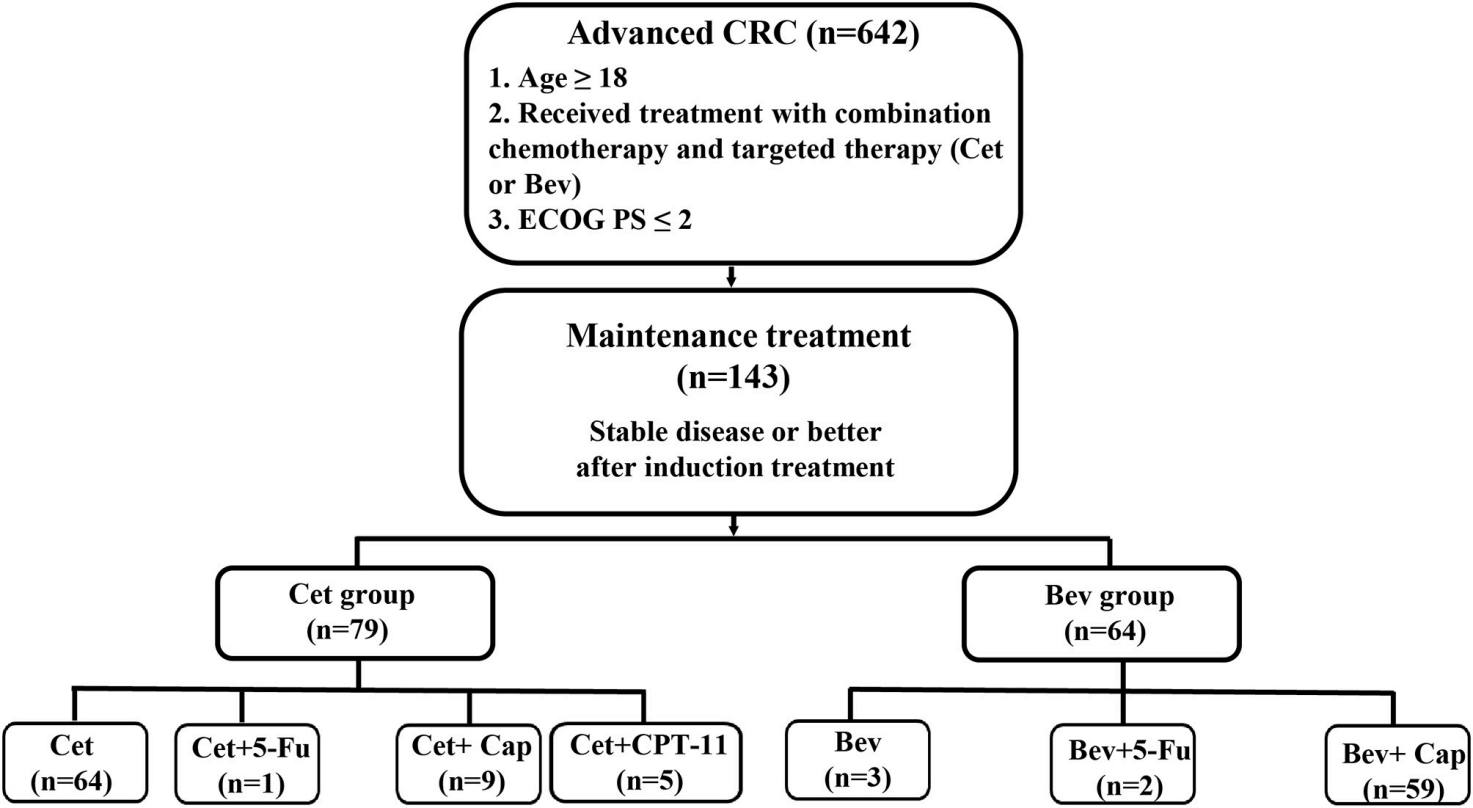
Study design. Cap, Capecitabine; CPT-11, Irinotecan; Fu, fluorouracil.

After receiving approval from the Institutional Review Board, we reviewed the clinical records of all patients diagnosed with CRC who had been treated at the Second Affiliated Hospital of Zhejiang University School of Medicine between January 2010 and December 2018. We identified 5,495 patients with CRC, of whom 642 patients were treated with combination chemotherapy and targeted therapy (Cet or Bev). Patients eligible for inclusion met the following criteria: (1) were older than 18 years; (2) had histologically proven CRC; and (3) had stable disease or better after induction treatment (chemotherapy combined with Cet or Bev) and accepted maintenance therapy with Cet or Bev. Finally, a total of 143 patients were eligible for inclusion in the study (Figure 1).

### Recording and Definitions of Variables

For each patient, the following data were recorded: age, sex, TNM stage, date of diagnosis, treatment, date of death or last known follow-up, information about primary tumor site and metastatic sites, and so on. Maintenance therapy was defined as patients who had stable disease or better after chemotherapy combined with targeted drugs who received continuous treatment with low-intensity, low-toxicity drugs for more than one cycle regardless of the line of the induction treatment regimen. PFS was defined as the time from the beginning of maintenance therapy to progressive disease (PD), death, the date of the last follow-up visit, or the time of the modification of treatment strategies. The duration of maintenance therapy was defined as the time from the beginning of maintenance therapy to the last maintenance therapy session.

### Statistical Analyses

Continuous variables were compared using the Mann-Whitney test, and categorical variables were compared with Fisher's exact or the Chi-square test. PFS and the duration of maintenance therapy were analyzed with the Kaplan-Meier method and compared using the two-sided log-rank test. Hazard ratios (HRs) and the corresponding 95% confidence intervals (CIs) were estimated with the Cox multivariate regression model. The 12-month PFS rate was compared using the *Z* test. We performed all analyses with GraphPad Prism version 8.0 (GraphPad Software, Inc.) and SPSS version 22.0 (SPSS, Inc.).

## Results

### Patient Characteristics

A total of 143 patients were eligible for inclusion in the study, including 55 females (38.5%) and 88 males (61.5%). The median age of the patients at the start of maintenance treatment was 62 years. Among those patients, 79 (55.2%) had maintenance treatment with Cet, while 64 (44.8%) had maintenance treatment with Bev. In the Cet group, all patients had KRAS wild-type and 50 (63.3%) patients had NRAS wild-type. As shown in Table 1, the baseline characteristics were well-balanced between the two groups, except for the higher percentage of patients with a left-sided primary tumor in the Cet group than in the Bev group (86.1 vs. 62.5%, *P* < 0.0001).

**Table 1:** Baseline patient characteristics.

	**Maintenance therapy regimen**			
**Variable**	**Cetuximab**	**Bevacizumab**		**All**
	***n* = 79 (55.2%)**	***n* = 64 (44.8%)**	***P*-value**	***n* = 143 (100%)**
Gender			0.24	
Female	27 (34.2%)	28 (43.8%)		55(38.5%)
Male	52 (65.8%)	36 (56.3%)		88 (61.5%)
Age at maintenance, median (range)	62.0 (21.0–90.0)	61.0 (24.0–76.0)	0.70	62.0 (21.0–90.0)
Smoking			0.21	
Yes	34 (43.0%)	21 (32.8%)		55 (38.5%)
No	45 (57.0%)	43 (67.2%)		88 (61.5%)
Primary tumor site			0.01	
Right colon	11 (13.9%)	24 (37.5%)		35 (24.5%)
Left colon	68 (86.1%)	40 (62.5%)		108 (75.5%)
Number of metastatic sites at start of maintenance therapy			0.16	
0	0 (0.0%)	2 (3.1%)		2 (1.4%)
1	35 (44.3%)	18 (28.1%)		53 (37.1%)
2	28 (35.4%)	25 (39.1%)		53 (37.1%)
≥3	14 (17.7%)	16 (25.0%)		30 (21.0%)
Unknown	2 (2.5%)	3 (4.7%)		5 (3.5%)
Metastatic time			0.49	
Synchronous	51 (64.6%)	39 (60.9%)		90 (62.9%)
Metachronous	26 (32.9%)	25 (39.1%)		51 (35.7%)
Unknown	2 (2.5%)	0 (0.0%)		2 (1.4%)
Preoperative chemotherapy			0.10	
Yes	20 (25.3%)	9 (14.1%)		29 (20.3%)
No	59 (74.7%)	55 (85.9%)		114 (79.7%)
Surgical resection of primary tumor			0.16	
Yes	59 (74.7%)	54 (84.4%)		113 (79.0%)
No	20 (25.3%)	10 (15.6%)		30 (21.0%)
Surgical resection of metastatic sites			0.83	
Yes	26 (32.9%)	20 (31.3%)		46 (32.2%)
No	53 (67.1%)	44 (68.8%)		97 (67.8%)
Simultaneous resection			0.80	
Yes	10 (12.7%)	10 (15.6%)		20 (14.0%)
No	68 (86.1%)	54 (84.4%)		122 (85.3%)
Unknown	1 (1.3%)	0 (0.0%)		1 (0.7%)
Line of maintenance therapy initiated			0.68	
1	34 (43.0%)	23 (35.9%)		57 (39.9%)
2	31 (39.2%)	29 (45.3%)		60 (42.0%)
≥3	14 (17.7%)	12 (18.8%)		26 (18.1%)

*simultaneous resection refers to whether metastases and primary tumor site are simultaneously resected*.

### Treatment Efficacy

**Figure 2:**
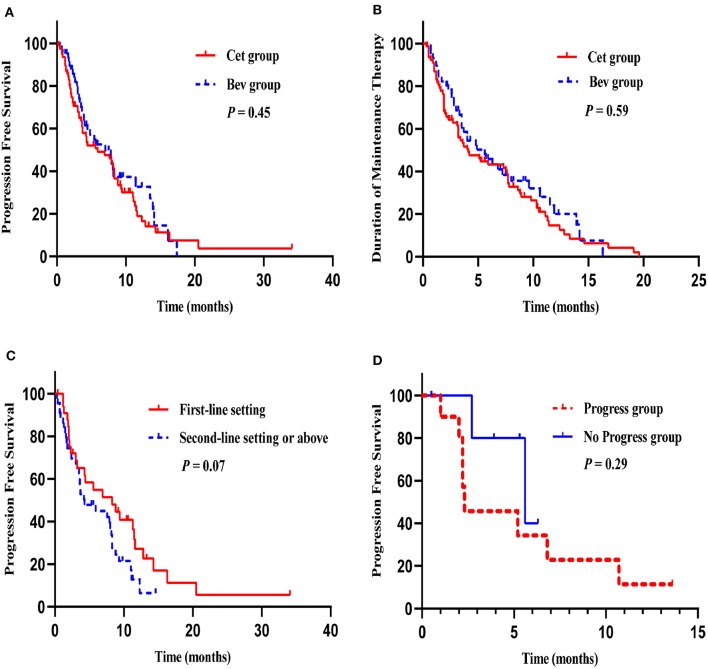
Survival curves. PFS in the Cet group and Bev group **(A)**, The median duration of maintenance therapy in the Cet group and Bev group **(B)**, PFS of maintenance therapy after first-line induction chemotherapy and second-line chemotherapy or above in the Cet group **(C)**, PFS of maintenance therapy in progress group and no progress group after the reintroduction of the original plan **(D)**.

The median duration of follow-up was 15.2 months (range: 3.5–87.5 months). The median PFS in the Cet group and Bev group were 5.9 months (95% CI 2.30–9.50) and 7.0 months (95% CI 3.69–10.31), respectively (HR 1.17, 95% CI 0.77–1.79; *P* = 0.45) (Figure 2A). The 12-month PFS rate was 18.9% in the Cet group and 32.3% in the Bev group (*P* = 0.15). The median duration of maintenance therapy was 4.0 months (95% CI 1.94–5.99) in the Cet group and 4.8 months (95% CI 2.68–6.98) in the Bev group (HR 0.90, 95% CI 0.61–1.33; *P* = 0.59) (Figure 2B).

Maintenance treatment was initiated following a first-line setting (39.9%) or second-line setting or above (60.1%); there were no significant differences between the two groups (*P* = 0.68). The most commonly used induction chemotherapy regimens were FOLFIRI (48.1 vs. 51.6%), FOLFOX (40.5 vs. 25.0%), and XELOX (1.3 vs. 15.6%) in the Cet group and the Bev group. In the Cet group, the median PFS with maintenance therapy after first-line induction chemotherapy and second-line chemotherapy or above were 8.3 months (95% CI 2.56–14.11) and 4.3 months (95% CI 1.61–6.99), respectively (HR 1.64, 95% CI 0.95–2.82; *P* = 0.07) (Figure 2C). In the Bev group, the median PFS with maintenance therapy after first-line induction chemotherapy and second-line chemotherapy or above were 5.6 months (95% CI 0.00–11.27) and 7.0 months (95% CI 2.46–11.55), respectively (HR 1.66, 95% CI 0.75–3.67; *P* = 0.21).

### Analyses of Multiple Maintenance Treatments

In our study, 21 patients received maintenance therapy twice, and subgroup analyses showed that the median PFS for the first maintenance therapy and the second maintenance therapy were 3.2 months (95% CI 1.69–4.78) and 5.2 months (95% CI 1.58–8.83), respectively (HR 0.89, 95% CI 0.44–1.81; *P* = 0.75). Among these patients, 16 patients underwent reintroduction of the original plan. 5 patients received Cet maintenance therapy following Bev maintenance therapy, and compared with the remaining 11 patients who received the same drug (Cet or Bev) in two maintenance therapies, the median PFS was 5.6 months (95% CI 1.43–9.77) vs. 2.3 months (95% CI 2.11–2.49) (HR 0.43, 95% CI 0.09–2.15; *P* = 0.29) (Figure 2D).

### Univariate and Multivariate Analyses

The factors that impacted PFS and the duration of maintenance therapy in the univariate analyses are shown in Tables 2, 3. Then, we performed a multivariate analysis that depended on the outcomes of the univariate analyses. In the Cet group, the number of metastatic sites at the start of maintenance ≥2 (vs. 1) was associated with the significantly worst prognosis, and the resection of metastatic sites had a positive impact on PFS (Table 4). In the Bev group for PFS, resection of the primary tumor had a positive impact on survival, while metachronous metastasis was associated with a significantly worse prognosis. The resection of the primary tumor had a positive impact on survival, and ≥3 lines of maintenance therapy initiated (vs. 1) had a negative effect on survival for the duration of maintenance therapy.

**Table 2:** Univariate analyses for PFS and duration of maintenance therapy in Cet group.

		**PFS**		**Duration of maintenance therapy**	
**Factors**		**95% CI**	***P*-value**	**95% CI**	***P*-value**
Age at maintenance					
	<62	1.00 (reference)		1.00 (reference)	
	≥62	1.21 (0.72–2.04)	0.47	1.01 (0.62–1.64)	0.99
Gender					
	Female	1.00 (reference)		1.00 (reference)	
	Male	0.74 (0.43–1.27)	0.27	0.67 (0.40–1.13)	0.14
Smoking					
	No	1.00 (reference)		1.00 (reference)	
	Yes	1.20 (0.71–2.02)	0.50	1.53 (0.92–2.54)	0.11
Primary tumor site					
	Left colon	1.00 (reference)		1.00 (reference)	
	Right colon	0.63 (0.30–1.35)	0.24	0.52 (0.24–1.09)	0.24
Resection of primary tumor					
	No	1.00 (reference)		1.00 (reference)	
	Yes	0.90 (0.49–1.65)	0.74	1.01 (0.57–1.81)	0.97
Preoperative chemotherapy					
	No	1.00 (reference)		1.00 (reference)	
	Yes	0.96 (0.54–1.71)	0.89	0.88 (0.51–1.52)	0.65
Simultaneous resection					
	No	1.00 (reference)		1.00 (reference)	
	Yes	0.64 (0.29–1.42)	0.27	0.84 (0.12–6.10)	0.86
Resection of metastatic sites					
	No	1.00 (reference)		1.00 (reference)	
	Yes	0.62 (0.35–1.08)	0.09	0.66 (0.39–1.10)	0.11
Metastatic time					
	Synchronous	1.00 (reference)		1.00 (reference)	
	Metachronous	1.18 (0.67–2.08)	0.56	1.26 (0.75–2.13)	0.38
Number of metastatic sites at start of maintenance therapy					
	1	1.00 (reference)		1.00 (reference)	
	2	2.20 (1.19–4.06)	0.01	2.12 (1.17–3.84)	0.01
	≥3	2.39 (1.14–5.01)	0.02	2.39 (1.14–5.01)	0.01
Line of maintenance therapy initiated					
	1	1.00 (reference)		1.00 (reference)	
	2	1.95 (1.06–3.56)	0.03	1.66 (0.94–2.94)	0.08
	≥3	1.26 (0.61–2.59)	0.54	1.45 (0.75–2.81)	0.27
Maintenance therapy regimen					
	Single therapy	1.00 (reference)		1.00 (reference)	
	Combination chemo	1.43 (0.77–2.66)	0.25	1.16 (0.63–2.13)	0.64

*PFS, progression-free survival; CI, confidence intervals; chemo, chemotherapy*.

**Table 3:** Univariate analyses for PFS and duration of maintenance therapy in Bev group.

		**PFS**		**Duration of maintenance therapy**	
**Factors**		**95% CI**	***P*-value**	**95% CI**	***P*-value**
Age at maintenance					
	<61	1.00 (reference)		1.00 (reference)	
	≥61	1.25 (0.63–2.45)	0.53	1.25 (0.67–2.34)	0.49
Gender					
	Female	1.00 (reference)		1.00 (reference)	
	Male	1.54 (0.75–3.17)	0.24	1.58 (0.83–3.03)	0.17
Smoking					
	No	1.00 (reference)		1.00 (reference)	
	Yes	1.16 (0.56–2.42)	0.69	1.19 (0.61–2.32)	0.61
Primary tumor site					
	Left colon	1.00 (reference)		1.00 (reference)	
	Right colon	0.78 (0.38–1.62)	0.51	0.79 (0.41–1.54)	0.49
Resection of primary tumor					
	No	1.00 (reference)		1.00 (reference)	
	Yes	0.21 (0.08–0.56)	0.02	0.13 (0.06–0.32)	0.00
Preoperative chemotherapy					
	No	1.00 (reference)		1.00 (reference)	
	Yes	0.99 (0.40–2.42)	0.98	1.08 (0.48–2.44)	0.86
Simultaneous resection					
	No	1.00 (reference)		1.00 (reference)	
	Yes	0.26 (0.06–1.09)	0.07	0.33 (0.10–1.08)	0.07
Resection of metastatic sites					
	No	1.00 (reference)		1.00 (reference)	
	Yes	0.38 (0.17–0.85)	0.02	0.32 (0.14–0.69)	0.04
Metastatic time					
	Synchronous	1.00 (reference)		1.00 (reference)	
	Metachronous	1.60 (0.81–3.18)	0.18	1.28 (0.69–2.38)	0.43
Number of metastatic sites at start of maintenance therapy					
	0	1.00 (reference)		1.00 (reference)	
	1	0.49 (0.11–2.32)	0.37	0.46 (0.10–2.09)	0.31
	2	0.53 (0.12–2.39)	0.41	0.69 (0.16–3.00)	0.62
	≥3	0.39 (0.08–1.87)	0.24	0.37 (0.08–1.76)	0.21
Line of maintenance therapy initiated					
	1	1.00 (reference)		1.00 (reference)	
	2	1.44 (0.62–3.35)	0.40	1.17 (0.56–2.48)	0.68
	≥3	2.31 (0.88–6.10)	0.09	1.85 (0.78–4.41)	0.17
Maintenance therapy regimen					
	Single therapy	1.00 (reference)		1.00 (reference)	
	Combination chemo	0.60 (0.14–2.59)	0.49	0.53 (0.16–1.78)	0.30

*PFS, progression-free survival; CI, confidence intervals; chemo, chemotherapy*.

**Table 4:** Multivariate analyses for PFS and duration of maintenance therapy in Cet group.

	**PFS**		**Duration of maintenance therapy**	
**Factors**	**95% CI**	***P*-value**	**95% CI**	***P*-value**
Resection of metastatic sites				
No	1.00 (reference)		–	
Yes	0.48 (0.26–0.89)	0.02	–	–
Number of metastatic sites at start of maintenance therapy				
1	1.00 (reference)		–	
2	2.19 (1.19–4.03)	0.01	–	–
≥3	3.24 (1.48–7.09)	0.003	–	–
**Multivariate analyses for PFS and duration of maintenance therapy in Bev group**				
Resection of primary tumor				
No	1.00 (reference)		1.00 (reference)	
Yes	0.14 (0.05–0.42)	<0.0001	0.08 (0.03–0.22)	<0.0001
Metastatic time				
Synchronous	1.00 (reference)		–	
Metachronous	2.23 (1.04–4.79)	0.04	–	–
Line of maintenance therapy initiated				
1	–	–	1.00 (reference)	
2	–	–	1.63 (0.74–3.58)	0.22
≥3	–	–	3.59 (1.35–9.54)	0.01

*PFS, progression-free survival; CI, confidence intervals; chemo, chemotherapy*.

### Safety

Our study suggested that the incidence of adverse events during maintenance therapy was lower than that during induction chemotherapy, which is shown in Table 5. The incidences of hematological toxicity and skin rash were higher in the Cet group than in the Bev group. The incidence of hypertension was higher in the Bev group than in the Cet group. There were no significant differences between the two groups. During maintenance therapy, the most common adverse events were grades 1–2, and the frequency of any grade 3 adverse events was low. In addition, there were no grade 4 adverse events. The most common grade 3 adverse events on maintenance therapy were anemia (3 [3.8%] in the Cet group vs. 0 [0.0%] in the Bev group), neutropenia (0 [0.0%] vs. 2 [3.2%]), hypertension (0 [0.0%] vs. 3 [4.8%]) and abnormal liver function (1 [1.3%] vs. 1 [1.6%]).

**Table 5:** Adverse events considered relevant to treatment.

	**Induction phase**			**Maintenance phase**		
**Adverse events**	**Cet group** ***n* = 78 (54.5%)**	**Bev group** ***n* = 63 (44.1%)**	***P*-value**	**Cet group** ***n* = 78 (54.5%)**	**Bev group** ***n* = 63 (44.1%)**	***P*-value**
Leucopenia			0.74			–
Grade 1–2	54 (69.2)	37 (58.7)		16 (20.5)	11 (17.5)	
Grade 3–4	5 (6.4)	5 (7.9)		0 (0.0)	0 (0.0)	
Anemia			1.00			0.09
Grade 1–2	22 (28.2)	17 (27.0)		7 (9.0)	11 (17.5)	
Grade 3–4	2 (2.6)	1 (1.6)		3 (3.8)	0 (0.0)	
Neutropenia			0.55			0.16
Grade 1–2	35 (44.9)	28 (44.4)		15 (19.2)	9 (14.3)	
Grade 3–4	24 (30.8)	15 (23.8)		0 (0.0)	2 (3.2)	
Thrombocytopenia			1.00			–
Grade 1–2	16 (20.5)	14 (22.2)		6 (7.7)	13 (20.6)	
Grade 3–4	1 (1.3)	0 (0.0)		0 (0.0)	0 (0.0)	
Hypertension			0.11			0.20
Grade 1–2	17 (21.8)	16 (25.4)		7 (9.0)	5 (7.9)	
Grade 3–4	1 (1.3)	6 (9.5)		0 (0.0)	3 (4.8)	
Abnormal liver function			1.00			1.00
Grade 1–2	52 (66.7)	37 (58.7)		25 (32.1)	16 (25.4)	
Grade 3–4	1 (1.3)	1 (1.6)		1 (1.3)	1 (1.6)	

**Table:** 

	**Induction phase**		**Maintenance phase**	
**Grade1–4 adverse events**	**Cet group** ***n*** **=** **78 (54.5%)**	**Bev group** ***n*** **=** **63 (44.1%)**	**Cet group** ***n*** **=** **78 (54.5%)**	**Bev group** ***n*** **=** **63 (44.1%)**
Asthenia	65 (83.3)	52 (82.5)	42 (53.8)	31 (49.2)
Alopecia	46 (59.0)	38 (60.3)	19 (24.4)	18 (28.6)
Nausea	50 (64.1)	37 (58.7)	8 (10.3)	1 (1.6)
Vomiting	34 (43.6)	25 (39.7)	5 (6.4)	0 (0.0)
Diarrhea	18 (23.1)	14 (22.2)	2 (2.6)	4 (6.3)
Hand-foot syndrome	13 (16.7)	8 (12.7)	15 (19.2)	13 (20.6)
Skin rash	26 (33.3)	1 (1.6)	17 (21.8)	0 (0.0)
Mucositis	7 (9.0)	2 (3.2)	1 (1.3)	4 (6.3)

## Discussion

This retrospective study indicated that the median PFS was similar between the Cet and Bev groups and that the incidence of adverse events during maintenance therapy was lower than that during induction chemotherapy, suggesting that maintenance therapy with Cet or Bev can be safely and effectively incorporated into treatment with intermittent chemotherapy.

Most clinical trials have shown that maintenance therapy with Bev could result in survival benefits without compromising the quality of life (9, 16, 17, 19, 20). At present, maintenance therapy with Bev has become a standard strategy in advanced CRC. The CAIRO3 trial showed that the median PFS2 was 11.7 months in the maintenance therapy with Bev plus capecitabine arm and 8.5 months in the observation arm, and the difference was statistically significant (HR 0.67; 95% CI 0.56–0.81; *P* < 0.0001) (9). The Stop and Go trial showed that the median PFS was 11.0 months in the maintenance therapy with Bev plus capecitabine group and 8.3 months in the observation arm, and the difference was statistically significant (HR 0.6; *P* = 0.002) (16). In MACRO, although non-inferiority could not be confirmed, this study suggested that maintenance therapy with Bev might be an appropriate option following induction chemotherapy (20). In our study, the median PFS of the Bev group was 7.0 months. There seems to be no significant difference between our results and the results of the abovementioned clinical trials.

For RAS and BRAF wild-type left-sided advanced CRC patients, chemotherapy with an anti-EGFR agent is the standard first-line treatment. Although not extensively studied, maintenance therapy with Cet in advanced CRC has been suggested (12, 21, 22). Although the COIN-B trial did not conduct statistical analyses, maintenance therapy with Cet improved PFS and OS with lower toxicity in mCRC patients (22). The NORDIC-VII, NORDIC-7.5, and MACRO-2 TTD studies evaluated maintenance therapy with Cet weekly or biweekly vs. continuous chemotherapy in mCRC patients and suggested that maintenance therapy with Cet was safely integrated into treatment with intermittent chemotherapy and might have contributed to a longer chemotherapy-free interval (12, 21, 23). However, there was insufficient evidence regarding maintenance therapy with Cet. In our study, patients received drug donations for maintenance treatment with Cet in clinical practice via to the patient assistance programs in China. The median PFS was 5.9 months in the Cet group and 7.0 months in the Bev group (HR 1.17, 95% CI 0.77–1.79; *P* = 0.45). Although the median PFS of the Bev group was longer than that of the Cet group, the difference was non-significant. In our study, the majority of patients in the Bev group received combination chemotherapy as maintenance therapy, while the Cet group received Cet monotherapy, which might affect the efficacy of maintenance treatment. However, the univariate analyses showed that combination chemotherapy in the two groups was not associated with the prognosis compared with monotherapy. This was consistent with the available clinical trials, which suggested that the finding that Bev with capecitabine (or fluorouracil) could lead to more survival benefits than Bev monotherapy was controversial. The current conclusions were inconsistent. In addition, clinical trials of maintenance therapy with Cet all used Cet monotherapy as the comparison. Further research is needed. This finding indicated that maintenance therapy with Cet was similar to maintenance therapy with Bev and could be considered an appropriate option following induction chemotherapy for selected patients with advanced CRC.

In clinical trials, patients with advanced CRC usually receive maintenance therapy following first-line induction chemotherapy. However, in real-world clinical practice, some patients receive maintenance therapy following second-line chemotherapy or above. In the Cet group, the median PFS on maintenance therapy after first-line induction chemotherapy and second-line chemotherapy or above were 8.3 months and 4.3 months, respectively (HR 1.64, 95% CI 0.95–2.82; *P* = 0.07). Although there was no statistically significant difference, the PFS curves suggested that there could be a trend toward a better outcome for maintenance therapy after first-line induction therapy.

These trials showed that the median OS was longer with maintenance therapy, but no significant difference was found, except in the subgroup analyses of CAIRO3, which suggested that complete or partial response to induction treatment lead to a greater benefit from maintenance treatment than stable disease (9). However, the reason maintenance therapy does not provide OS benefits remains unclear. Nevertheless, with the increasing improvement of therapeutic effects and treatment strategies, the correlation between PFS and OS worsens.

In our study, among the 21 patients who received two maintenance treatments, a subgroup analysis showed that the second maintenance treatment still provided a PFS benefit, and no statistically significant differences were found compared with the first maintenance treatment. The results indicated that CRC patients could receive multiple maintenance therapies. However, because the endpoint of OS was not met, we could not further explore whether multiple maintenance therapies could benefit OS. Further research is needed to explore this issue.

RAS mutations are a negative predictor of prognosis in patients with mCRC receiving the anti-EGFR antibody (24). However, molecular testing of the RAS gene is extremely limited because tumor tissue is limited, and biopsies are invasive. More recently, some studies suggested that the consistency of liquid biopsy and tumor-tissue biopsy testing was more than 90% in the detection of the RAS gene (25, 26). Liquid biopsy is an important method of monitoring RAS mutation status in mCRC patients.

In our study, five patients received Cet maintenance therapy following Bev maintenance therapy because they progressed after the reintroduction of the original plan. Although there was no statistically significant difference, the PFS curves (Figure 2D) suggested a trend toward a relatively better outcome in patients who received Cet maintenance therapy following Bev maintenance therapy. This finding seemed to be explained by recent studies. Diaz et al. (27) and Siravegna et al. (28) found that in KRAS wild-type mCRC patients, KRAS mutation gradually increased during treatment with anti-EGFR antibody and gradually decreased after stopping treatment with anti-EGFR antibody or switching to other targeted drugs. The half-life of RAS mutations was 3.4 months (29). Siena et al. also found that the analysis of plasma samples showed that the first detected emergence of RAS mutations occurred a median of 3.6 months (range, 0.3–7.5 months) earlier than imaging progression. In addition, those who had emerging RAS mutations at progression had a similar median PFS to those patients who remained wild-type (29). These results suggested that except RAS mutations led to resistance to anti-EGFR antibodies, other mechanisms led to disease progression. The dynamic monitoring of the RAS gene status could predict acquired resistance to anti-EGFR antibodies and provide evidence for the adjustment of treatment strategies. During maintenance treatment with Cet, the efficacy, and prognosis could be predicted based on the regular detection of the status of the RAS gene, and the optimal timing of rechallenge with Cet could therefore be estimated. However, the threshold of RAS mutations and the time interval of anti-EGFR antibody rechallenge remain controversial and deserve further study.

Our study found that resection of the primary site was significantly associated with both PFS and the duration of maintenance therapy in the Bev group. This finding was consistent with the findings of the CAIRO3 study, which suggested that resection of the primary site was beneficial in patients with CRC. For the Cet group, the number of metastatic sites at the start of maintenance was significantly associated with PFS. Additionally, subgroup analyses indicated that there was a trend toward a better outcome for maintenance therapy after first-line induction therapy. Therefore, maintenance therapy with Cet following first-line induction chemotherapy seemed to result in greater survival benefits in CRC patients.

Another major goal of maintenance therapy was to reduce the toxicity of continuous chemotherapy and improve the quality of life of mCRC patients. Because of the limitations of the retrospective study design, we could not evaluate the adverse events of induction chemotherapy and maintenance therapy in detail. However, our study suggested that the incidence of adverse events during maintenance therapy was lower than that during induction chemotherapy. During maintenance therapy, the most common adverse events were grades 1–2, and the frequency of any grade 3 adverse events was low. In addition, there were no grade 4 adverse events. The results indicated that maintenance therapy was safe and tolerable, which is consistent with the results in clinical trials (12, 16, 20, 23).

## Conclusion

Our study suggests that maintenance therapy with Cet or Bev can be considered an appropriate option following induction chemotherapy for selected patients with advanced CRC and that patients can receive multiple maintenance therapies. Maintenance therapy with Cet after first-line induction chemotherapy seems to result in relatively greater survival benefits. However, more studies are needed to confirm these findings.

## Data Availability Statement

The raw data supporting the conclusions of this article will be made available by the authors, without undue reservation, to any qualified researcher.

## Ethics Statement

The studies involving human participants were reviewed and approved by Human Research Ethics Committee of the Second Affiliated Hospital, Zhejiang University School of Medicine. The ethics committee waived the requirement of written informed consent for participation.

## Author Contributions

YY made substantial contributions to the conception and design of the work. HH and XL acquired the clinical data and drafted the manuscript. WC revised it critically for important intellectual content. DW and JX interpreted the data.

## Conflict of Interest

The authors declare that the research was conducted in the absence of any commercial or financial relationships that could be construed as a potential conflict of interest.
